# A 4.6 Mb Inversion Leading to *PCDH15*-*LINC00844* and *BICC1*-*PCDH15* Fusion Transcripts as a New Pathogenic Mechanism Implicated in Usher Syndrome Type 1

**DOI:** 10.3389/fgene.2020.00623

**Published:** 2020-07-02

**Authors:** Christel Vaché, Jacques Puechberty, Valérie Faugère, Floriane Darmaisin, Alessandro Liquori, David Baux, Catherine Blanchet, Gema Garcia-Garcia, Isabelle Meunier, Franck Pellestor, Michel Koenig, Anne-Françoise Roux

**Affiliations:** ^1^Laboratoire de Génétique Moléculaire, CHU de Montpellier, Université de Montpellier, Montpellier, France; ^2^Service de Génétique Clinique, CHU de Montpellier, Montpellier, France; ^3^Service ORL, CHU de Montpellier, Montpellier, France; ^4^Centre de Référence Maladies Sensorielles Génétiques, CHU de Montpellier, Université de Montpellier, Montpellier, France; ^5^Laboratoire de Génétique Chromosomique, Plateforme ChromoStem, CHU de Montpellier, Montpellier, France

**Keywords:** Usher syndrome, *PCDH15*, paracentric inversion, fusion transcripts, long-read sequencing

## Abstract

Usher type 1 syndrome is a rare autosomal recessive disorder involving congenital severe-to-profound hearing loss, development of vision impairment in the first decade, and severe balance difficulties. The *PCDH15* gene, one of the five genes implicated in this disease, is involved in 8–20% of cases. In this study, we aimed to identify and characterize the two causal variants in a French patient with typical Usher syndrome clinical features. Massively parallel sequencing-based gene panel and screening for large rearrangements were used, which detected a single multi-exon deletion in the *PCDH15* gene. As the second pathogenic event was likely localized in the unscreened regions of the gene, *PCDH15* transcripts from cultured nasal cells were analyzed and revealed a loss of junction between exon 13 and exon 14. This aberration could be explained by the identification of two fusion transcripts, *PCDH15*-*LINC00844* and *BICC1*-*PCDH15*, originating from a 4.6 Mb inversion. This complex chromosomal rearrangement could not be detected by our diagnostic approach but was instead characterized by long-read sequencing, which offers the possibility of detecting balanced structural variants (SVs). This finding extends our knowledge of the mutational spectrum of the *PCDH15* gene with the first ever identification of a large causal paracentric inversion of chromosome 10 and illustrates the utility of screening balanced SVs in an exhaustive molecular diagnostic approach.

## Introduction

Usher syndrome (USH) is an autosomal recessive disorder characterized by bilateral sensorineural hearing loss (SNHL) and progressive deterioration of vision due to retinitis pigmentosa (RP). Vestibular dysfunction can be noted in a subset of patients. With an estimated prevalence that may be as high as 1/6,000 births (Kimberling et al., 2010), USH is the most common cause of hereditary deaf-blindness (Boughman et al., 1983). Among the three clinical USH types described according to the severity of hearing loss, the onset of RP, and the presence or absence of vestibular dysfunction, Usher type 1 (USH1) is the most severe form with congenital severe-to-profound SNHL with RP occurring in the first decade, and vestibular dysfunction.

To date, five causal genes: *MYO7A* (MIM#276903), *USH1C* (MIM#605242), *CDH23* (MIM#605516), *PCDH15* (MIM#605514), and *USH1G* (MIM#607696), and one conflicting gene, *CIB2* (MIM#605564) (Booth et al., 2018), have been associated with USH1. *PCDH15*, located on chromosome 10, spans nearly 1 Mb and encodes three prominent transmembrane protocadherin-15 (Pcdh15) splice isoforms (Pcdh15-CD1, Pcdh15-CD2, and Pcdh15-CD3) differing mainly in their cytoplasmic domains (CD). This gene is involved in 8% to 20% of USH1 cases (Roux et al., 2006; Le Guédard et al., 2007; Jiang et al., 2015; Ivanova et al., 2018; Sun et al., 2018) and presents a mutational spectrum comprising nonsense and splicing variants, out-of-frame insertions/deletions, some missense variants, and a significant proportion of large genomic rearrangements (see the Locus Specific Database LOVD^1^).

In a clinical context of laboratories offering academic expertise in molecular diagnosis of USH, the most commonly used approach is massively parallel sequencing (MPS) with gene panel analysis covering exonic and neighboring intronic sequences of USH genes. In addition, the study of large rearrangements and screening of deep intronic pathogenic variants are also performed when necessary (Roux et al., 2006; Vaché et al., 2012; Liquori et al., 2016). Although this combination of molecular methods identifies the great majority of USH genotypes, a few cases remain unresolved and require the introduction of new technologies to elucidate the pathogenic mechanisms thus far undetected.

In this study, using RNA assays and DNA long-read sequencing, we identified a large paracentric inversion causing USH1 in a patient. This pathogenic mechanism, never before linked to Usher syndrome, should be screened for patients with well-defined USH phenotype and incomplete genotype.

### Case Presentation

The proband (II-1, family S331), a French 27-year-old female, was investigated for both cochlear-vestibular and visual function in the French national reference center for genetic sensory disorders of Montpellier (MAOLYA).

**FIGURE 1:**
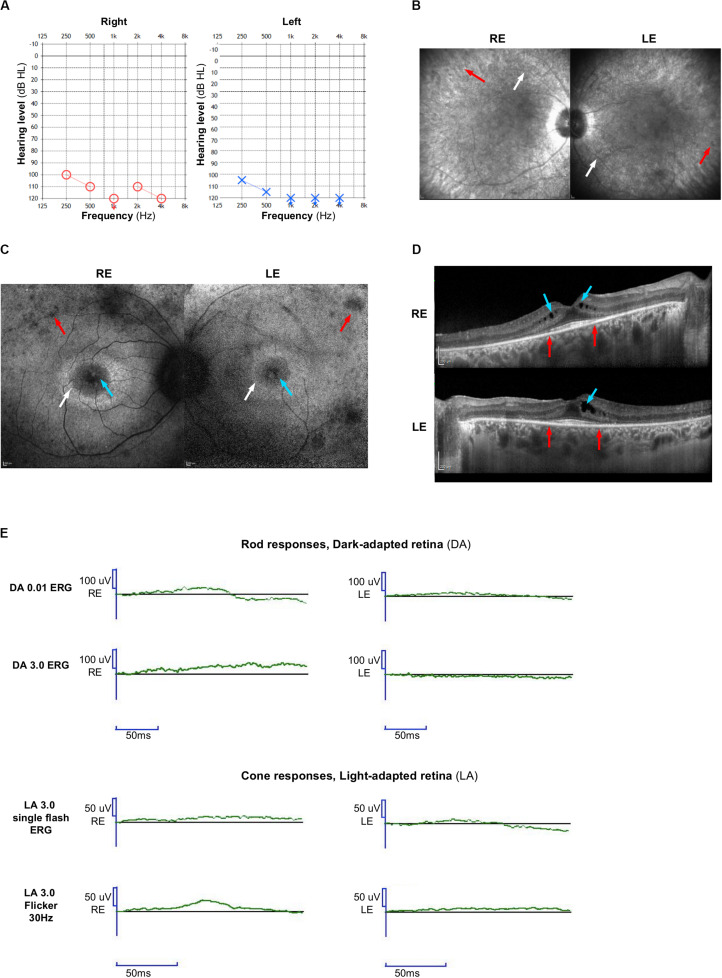
Clinical features. **(A)** Audiograms of the proband: blue circle indicates air conduction for right ear and red cross indicates air conduction for left ear. **(B)** On infrared reflectance frames, retinal vessel attenuation (white arrows), and few pigmentary changes along the vascular arcades (red arrows) are detected. **(C)** On fundus autofluorescence (FAF) photographs, foveal cysts are hyper-autofluorescent (blue arrows), whereas the peripheral retina is hypo-autofluorescent (red arrows). A typical annular perifoveal ring is disclosed in both eyes (white arrows). **(D)** On spectral-domain optical coherence tomography (SD-OCT) scans, note the complete disappearance of the ellipsoid zone, the interdigitation zone, and thinning of the outer nuclear layer (photoreceptors) except within the fovea (between red arrows). There is a macular edema in both eyes with cystic lesions mainly within the inner nuclear layer (blue arrows). **(E)** Full-field electroretinogram is similar to that seen in retinitis pigmentosa with severely reduced rod and cone responses. Right eye (RE); Left eye (LE).

Historic data reported a profound bilateral hearing loss diagnosed at nine months old. She was born before the national implementation of neonatal universal hearing screening. Her mother worried about her audition by the age of four months. Despite the important degree of hearing loss, the patient was fitted with behind-the-ear hearing aids all along her life, allowing environmental sounds perception. She used total communication. She was able to understand speech language using lip reading and cued speech and was taught speech language through speech therapy and school, but her intelligibility was poor. She could not use oral communication with strangers in everyday life, but used either sign language or written language. Independent walk was acquired at the age of two years old. The audiometric assessment confirmed a bilateral symmetric profound group three hearing loss on pure tone audiometry according to the 02/1bis recommendation of the Bureau International d’Audiophonologie (BIAP^2^). Air conduction without hearing aids is reported in Figure 1A. Otoscopy was normal. The vestibular assessment showed a bilateral areflexia in high and low frequencies (video head impulse test, rotational chair, and caloric videonystagmography). The vestibular score obtained from the computerized dynamic posturography sensory organization test was null; the visual score was decreased.

This patient reported poor night vision before the age of 10 years. At the same time, she was also clumsy and hit herself frequently. At the age of 27, her visual acuity was 7/10 in both eyes. Visual field was severely impaired with complete loss of the peripheral field, restricted to the 10–15 central degrees on Goldmann field. Infrared reflectance imaging (Figure 1B) detected retinal vessel attenuation that is a key sign of retinitis pigmentosa (white arrows) and pigmentary changes along the vascular arcades (red arrows). On blue light autofluorescence imaging (FAF, fundus autofluorescence frame), cystic foveal lesions were hyper-autofluorescent (Figure 1C, blue arrow), and outer retinal atrophy (loss of photoreceptors) appeared as small hypo-autofluorescent areas in the mid-peripheral retina (Figure 1C, red arrows). A typical annular perifoveal ring was also observed in both eyes (Figure 1C, white arrows). Spectral domain optical coherence tomography (Figure 1D) disclosed cystic foveal lesions (blue arrows) and remaining foveal ellipsoid zone (between red arrows). Outside the fovea, the outer nuclear layer, the ellipsoid zone, and the interdigitation zone lines disappeared. On full-field electroretinogram (Figure 1E), rod and cones responses were severely reduced.

Based on these observations a clinical diagnosis of Usher syndrome type I was established.

## Materials and Methods

### Consent and Ethics

Written informed consent to genetic testing was obtained from all participants prior to conducting investigations, and the local Ethics Committee approved the molecular analysis.

### Massively Parallel Sequencing and Large Rearrangements Screening

The DNA from the proband and her parents was isolated from peripheral blood samples using standard procedures and analyzed using an MPS-based gene panel approach on an Illumina MiSeq sequencer (Illumina, San Diego, CA, United States). The panel included 112 genes involved in USH, non-syndromic hearing loss and RP (García-García et al., 2016). The data analysis, resulting in the interpretation of the selected alterations, was performed according to our diagnosis workflow that particularly includes an in-house spreadsheet used to determine the copy number of each exon (Baux et al., 2017). The potential rearrangements were then validated by multiplex ligation-dependent probe amplification (MLPA) using the SALSA MLPA kit 292-A2 including 34 *PCDH15* specific probes (Supplementary Table S1) (MRC-Holland, Amsterdam, Netherlands) according to the manufacturer’s instructions.

### Nasal Epithelial Cell Culture and RNA Extraction

After local anesthesia (Xylocaïne 5% nébuliseur, Astrazeneca, France), nasals cells were obtained by gentle scraping of the lateral inferior turbinate of each nostril with ASI Rhino-Pro^®^ curettes (Arlington Scientific, Springville, UT, United States) and immediately suspended in serum-free bronchial epithelial cell growth medium (BEGM; Lonza, Basel, Switzeland) with 1X antibiotic-antimycotic (Thermo Fisher Scientific, Villebon sur Yvette, France). Cells were plated on 25-cm^2^ culture flasks coated with type I rat-tail collagen at 5 μg/cm^2^ (Thermo Fisher Scientific) and incubated at 37°C in 5% CO_2_. Once cells reached 80% confluency, total RNA was isolated using the Nucleo Spin^®^ RNA II isolation kit (Macherey-Nagel, Düren, Germany).

### RNA Analyses

For the analysis of *PCDH15* transcripts, total RNA was reverse transcribed using the SuperScript^TM^ III Reverse Transcriptase (Invitrogen, Carlsbad, CA, United States) and oligo(dT) primers, according to the manufacturer’s instructions. Five overlapping PCRs, F1 to F5, were then carried out with *PCDH15-*specific primers (Supplementary Table S2). Amplification products were purified and Sanger sequenced using the BigDye Terminator v1.1 cycle sequencing kit (Applied Biosystems, Warrington, United Kingdom) on a capillary ABI 3130xl Genetic Analyzer (Applied Biosystems) according to the manufacturers’ instructions.

For the 3′end amplification of the *PCDH15*-*LINC00844* fusion transcripts, total RNA was reverse transcribed using the SuperScript^TM^ III Reverse Transcriptase and the anchored oligo(dT) primer: 5′-ACTATCTAGAGCGGCCGC-oligo(dT)_22_-3′. Semi-nested PCRs were then performed with *PCDH15* forward primers (PCR1-exon10: 5′-CCATA TGCATCCTAGGACAG-3′; PCR2-exon11: 5′-CAACCATTTC GGACAGTCTC-3′) and a primer complementary with the anchored sequence of the oligo(dT)_22_ primer, and sequenced. The result was confirmed from cDNA synthesized with standard oligo(dT) and PCR amplified with specific *PCDH15* (exon10: 5′-CCATATGCATCCTAGGACAG-3′) and *LINC00844* (exon2: 5′-CTT CCA CAA AGA AAA TAT CAT GG-3′) primers, and sequenced.

For the 5′end amplification of *BICC1-PCDH15* fusion transcripts, 1 μg of total RNA was used as a template in the 5′-RACE cDNA synthesis reaction using the SMARTer^®^ RACE 5′ kit (Takara Bio Europe, Saint-Germain-en-Laye, France). The generated 5′-RACE-Ready cDNA samples were then amplified by nested PCRs with the universal long and short forward primers provided in the kit and reverse *PCDH15*-specific primers (PCR1-exon18: 5′-CACTATGTTTACTGTGGCAGTTGAGG-3′; PCR2-exon14: 5′-CCCTGGAGCGATGGTGATAAGCC-3′), and then Sanger sequenced. The result was confirmed from cDNA synthesized with oligo(dT) and PCR-amplified with specific *BICC1* (exon19: 5′-AGCTCTTTCAAAGGTTCTGAC) and *PCDH15* (exon19: 5′-TGTTGAATTGGTGAACACAGG-3′) primers, and sequenced.

### Long-Read Sequencing

For genomic long-read sequencing, genomic DNA was quantified with the Qubit dsDNA HS Assay Kit (Thermo Fisher Scientific, Illkirch, France) and used as the template for MinION library preparation using the ligation sequencing kit 1D (SQK-LSK109) according to the manufacturer’s instructions (Oxford Nanopore Technologies, Oxford, United Kingdom). Sequencing was performed using the Oxford Nanopore MinION sequencer on one R9.4.1/FLO-MIN106 flow cell. Datasets were converted from .fast5 to .fastq using Guppy basecaller software, and binary alignment map (BAM) files were created with Sambamba software (Tarasov et al., 2015) and loaded into the integrative genomics viewer (IGV) software (v 2.7.2) in order to align reads to the hg38 human reference genome (Robinson et al., 2011). Sequences of interest were selected and blasted against the NCBI nucleotide database using the blastn program. PCR amplifications were then performed with primers surrounding the detected *PCDH15*/*LINC00844* junction sequence (Forward: 5′-TGGTTTACTAGATATTTGACTTG-3′; Reverse: 5′-AGGCCTGAGCTCTGTAAGC-3′) and the *PCDH15* E18-26 deletion (Forward: 5′-AATGGATAGCCCTGAAAT CC-3′; Reverse: 5′-AAGACTGTATTTATGGACTAGAATGG-3′), and Sanger sequenced for validation.

For amplicon long-read sequencing, long-range PCR was carried out using the GoTaq^®^Long PCR Master Mix kit (Promega, Charbonnières-les-Bains, France), with primers designed to amplify the *BICC1*/*PCDH15* breakpoint (Forward: 5′-AAGATGGTTGCTCACTCACC-3′; Reverse: 5′-GACTTTA AAGCATTTCTTTTCAC-3′), following the manufacturer’s instructions. After purification, the 11 kb PCR product was sequenced with the MinIon system and reads were analyzed as previously described. The junction sequence was then validated by Sanger sequencing of the long-range PCR amplicon using internal primers.

Nomenclature of the variants follows the Human Genome Variation Society (HGVS) recommendations v19.01 (den Dunnen et al., 2016^3^) with nucleotide + 1 corresponding to the A of the ATG initiation codon in the *PCDH15* reference sequence NM_033056.3, NG_009191.2.

The variants reported in this study have been submitted to the public LOVD3 shared genetic database^4^.

## Results

### Identification of a Novel Large Deletion on the Maternal Allele

**FIGURE 2:**
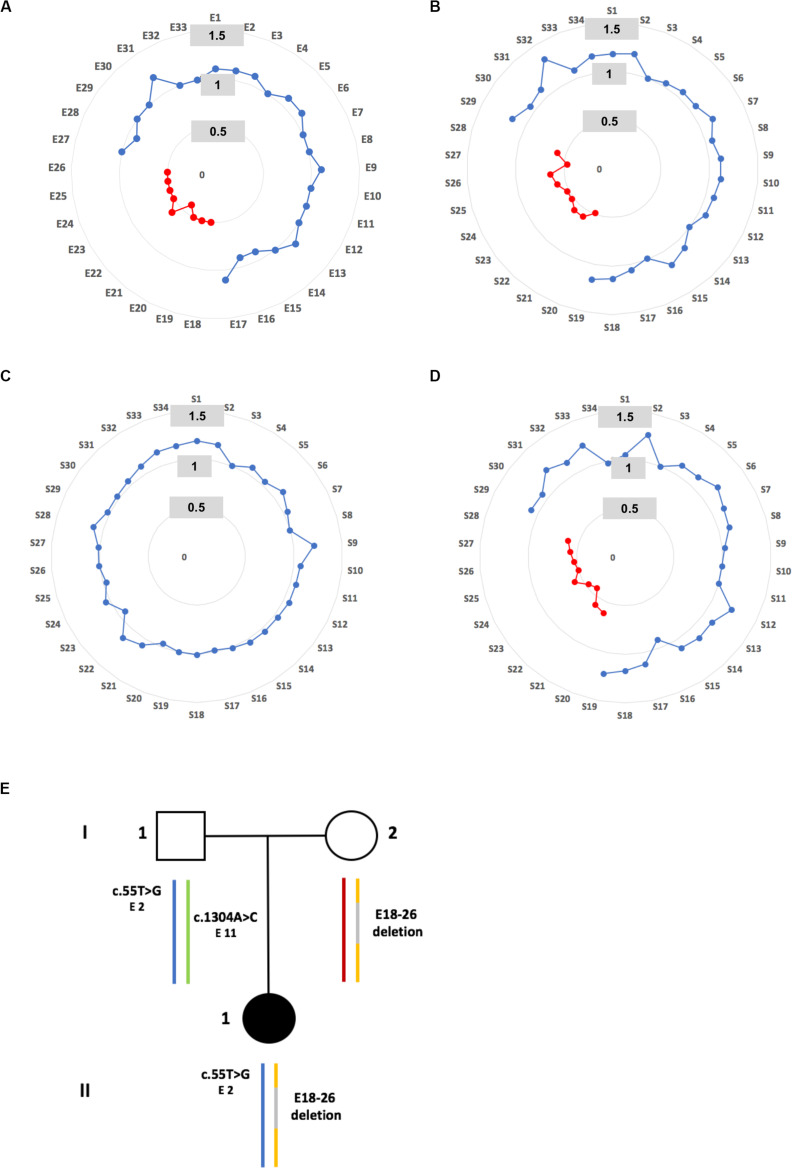
Identification of a maternally inherited deletion. **(A)** Graphic representation of relative copy number of *PCDH15* exons obtained by MPS for patient II.1. A normal value is defined between 0.7 and 1.2 and is represented by a blue spot, a heterozygous deletion is detected when values are lower than 0.7 and higher than 0.3, and is represented by a red spot. **(B)** Graphic representation of MLPA results for patient II.1. For each probe (S1 to S34), a ratio showing a range of 0.7–1.2 is classified as normal (blue spot), while a probe showing a ratio between 0.6 and 0.2 reflects a heterozygous deletion (red spot). **(C,D)** Graphic representations of MLPA results carried out in the father and the mother of patient II.1 respectively. **(E)** Pedigree of family S331. The blue and yellow colored vertical lines correspond to the inherited paternal and maternal allele respectively. A gray line indicates the deletion exon 18 to exon 26. The benign c.55T>G (exon 2) and c.1304A>G (exon 11) paternal substitutions, localized on different alleles, are point out.

Molecular diagnostic investigations of the 10 USH genes based on the MPS approach were performed in the family. Copy number variant analysis revealed a heterozygous exon 18 to exon 26 (E18-26) *PCDH15* deletion (Figure 2A). This damaging deletion has never before been found in our cohort of USH1 probands (*n* = 311) nor described in public population databases including the Locus Specific Database LOVD (LOVD^1^), the genome Aggregation Database (gnomAD^5^), the 1000 Genomes Project (GnomAD^6^), and the Exome Aggregation Consortium (ExAC^7^). This result was validated by MLPA (Figure 2B; relative probe signal comprised between 0.5 and 0.6 for probes S20 to S28 targeting exons 18 to 26), and a maternal inheritance was confirmed (Figures 2C–E).

As no other pathogenic candidate variant had been detected in the 63 NSHL genes and the 38 RP genes of our gene panel, we hypothesized that *PCDH15* was the causal gene carrying a paternal pathogenic variation in the unscreened intronic sequences leading to a splice defect.

### Detection of *PCDH15* Fusion Transcripts

**FIGURE 3:**
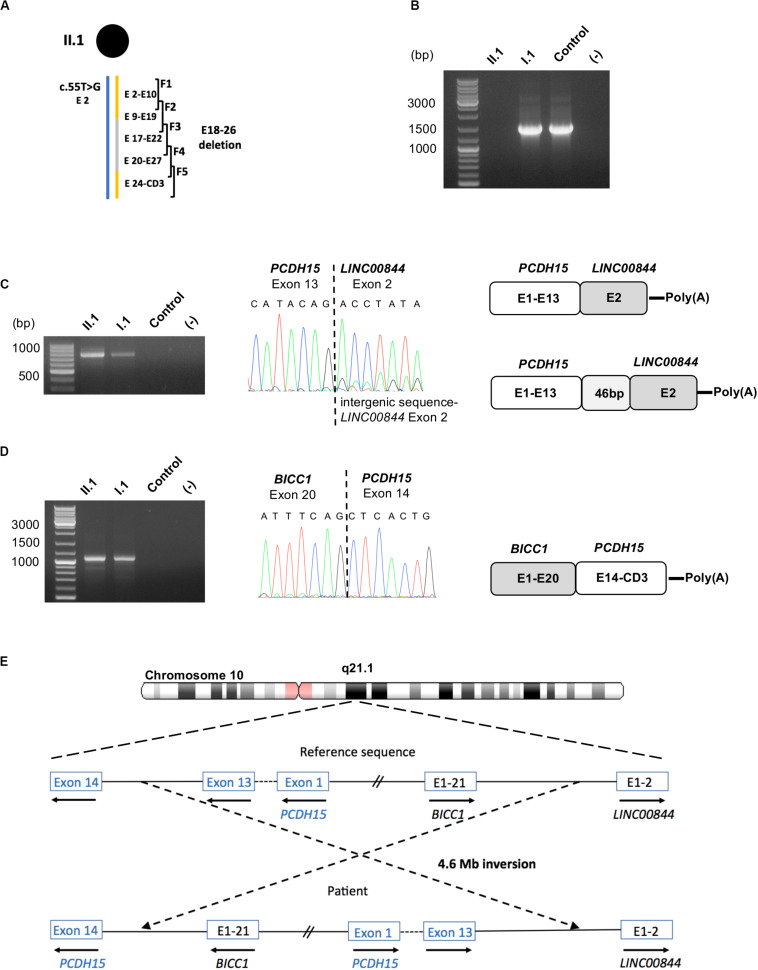
Identification of fusion transcripts. **(A)** Scheme of the five overlapping RT-PCRs (F1–F5) used in this study is presented above the patient II.1 haplotype. **(B)** RT-PCR amplification of *PCDH15* exon 9 to exon 19 from total RNA of patient II.1, her father I.1 and a control, showing an absence of amplicon for the patient. **(C)** Molecular characterization of the *PCDH15*-*LINC00844* transcripts: agarose gel electrophoresis of the RT-PCR products obtained from II.1, I.1 and a control; electrophoregram of the junction point between *PCDH15* exon 13 and *LINC00844* exon 2; schematic representation of the fusion transcripts. **(D)** Molecular characterization of the *BICC1*-*PCDH15* transcripts: agarose gel electrophoresis of the RT-PCR product obtained from II.1, I.1 and a control; electrophoregram of the junction point between *BICC1* exon 20 and *PCDH15* exon14; schematic representation of the fusion transcript. **(E)** Schematic representation of the hypothetic 4.6 Mb paracentric inversion leading to the synthesis of the identified fusion transcripts.

In order to highlight an aberrant splice event, total RNA of the patient, the father (I.1), and a control was isolated from nasal cells in culture (Vaché et al., 2010) and used as a template in five overlapping RT-PCR reactions (F1–F5) to analyze the coding sequence of the *PCDH15*-CD3 transcript (Figure 3A). Primers were designed to discriminate the two parental *PCDH15* alleles in amplicon F1, because of the benign heterozygous c.55T>G substitution absent in I.2. In addition, for amplicons F2–F5, at least one primer was localized in the deleted region on the maternal allele (E18–26 deletion). Visualization of the five RT-PCR products by agarose gel electrophoresis highlighted a lack of F2 amplification (exons 9–19, targeting the paternal allele) for II.1 and a specific c.1304A>C allele amplification in I.1 (Figure 3B). To define more precisely which exons were involved in this amplification defect, several RT-PCRs were conducted on the patient’s sample and revealed a systematic amplification failure when primer pairs were designed in order to generate fragments spanning the exons 13,14 junction.

In order to explain this result, complementary RNA analyses performed on II.1 and I.1, consisting of 3′ and 5′-amplification of cDNA ends, revealed the presence of the two fusion transcripts *PCDH15*-*LINC00844*, and *BICC1-PCDH15*, explaining our incapacity to amplify the exons 13,14 junction of the inherited paternal *PCDH15* transcripts. Indeed, the major *PCDH15*-*LINC00844* transcript (Figure 3C) was composed of *PCDH15* exons 1–13 followed by the second and last exon of the long intergenic non-protein coding RNA 844 (*LINC00844*), and the second transcript (Figure 3D) corresponded to a fusion between *BICC1* exons (minus the last coding exon 21) and *PCDH15* exons 14 to the last CD3-exon. As *PCDH15* is oriented in the opposite direction to *BICC1* and *LINC00844* in chromosome 10, we hypothesized that these fusion transcripts might be the indirect hallmarks of a large paracentric inversion of around 4.6 Mb (Figure 3E).

### Characterization of the Paracentric Inversion on the Paternal Allele

To confirm this hypothesis, DNA studies by MPS target enrichment were performed on II.1 to detect a potential breakpoint in the 30 kb *PCDH15* intron 13 sequence. This approach enabled us to analyze 74.3% of the sequence but failed to detect any aberrant event. The unscreened sequences were essentially repetitive DNA elements including a 5.8 kb human endogenous retrovirus subfamily H transposable element (HERVH). Based on this result we assumed that the breakpoint, occurring in repetitive sequence, has been missed by short read sequencing.

**FIGURE 4:**
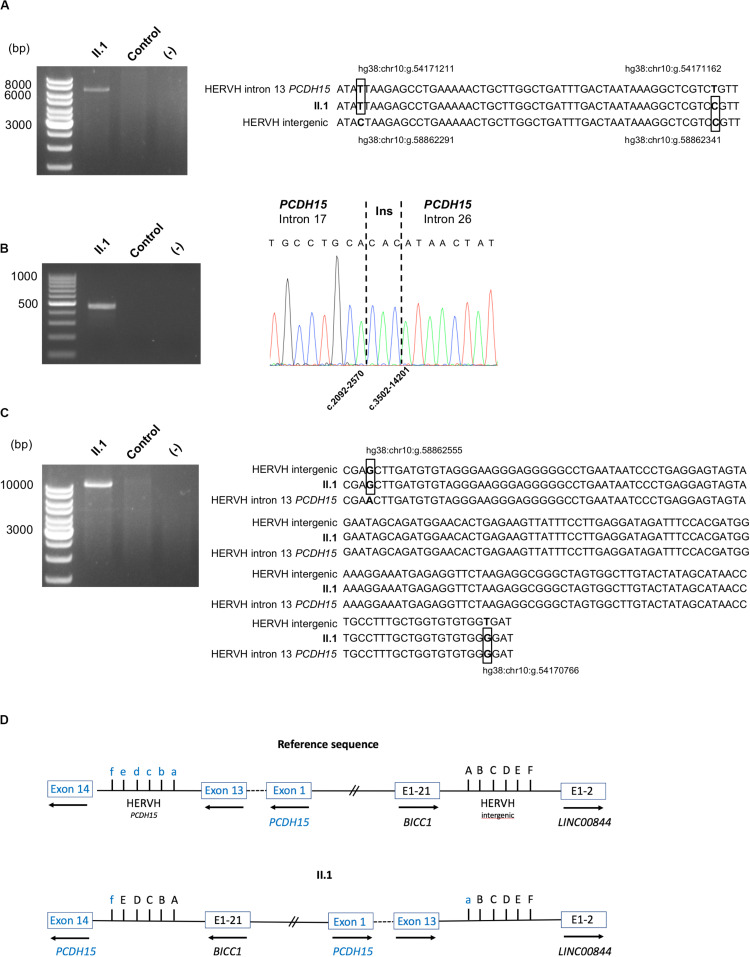
Characterization of the pathogenic alterations. **(A)** DNA validation of the paternal inherited inversion 3′ end junction: agarose gel electrophoresis of the PCR products obtained from patient II.1 and a control, using primers surrounding the junction; alignment of the HERVH reference sequences implicated in the 3′ end junction to the II.1 sequence. The divergent bases between the two HERVH reference sequences are in bold and boxed in the patient sequence. Genomic positions are indicated. **(B)** DNA validation of the maternal inherited deletion breakpoint: agarose gel electrophoresis of the PCR products obtained from II.1 and a control, using primers surrounding the junction; electrophoregram of the junction point between *PCDH15* intron 17 and *PCDH15* intron 26. **(C)** Characterization of the paternal inherited inversion 5′ end junction: agarose gel electrophoresis of the long-range PCR products obtained from II.1 and a control; alignment of the HERVH reference sequences implicated in the 5′ end junction to the II.1 sequence. The divergent bases between the two HERVH reference sequences are in bold and boxed in the patient sequence. Genomic positions are indicated. **(D)** Schematic representation of the large inversion detected in family S331. The divergent bases between the two HERVH reference sequences used for the description of the inversion using the HGVS 3′ rule are indicated: f, hg38:chr10:g.54170766; e, hg38: chr10:g.54170947; d, hg38:chr10:g.54171094; c, hg38:chr10:g.54171150; b, hg38:chr10:g.54171162; a, hg38:chr10:g.54171211; A, hg38:chr10:g. 58862291; B, hg38:chr10:g. 58862340; C, hg38:chr10:g. 58862352; D, hg38:chr10:g.58862408; E, hg38:chr10:g.58862555; F, hg38:chr10:g.58862736.

To characterize this structural variant (SV), genomic DNA obtained from the patient was then prepared and sequenced on a MinION sequencer (Oxford Nanopore Technologies) resulting in a total of 12 Gb output with a median read length of 25.5 kb. Visualization of the aligned sequence reads, using the Integrative Genomics Viewer (IGV) focused on the *PCDH15* intron 13, highlighted two reads with typical SV signatures (large clipped read tails). Analyses of these sequences, using the Blastn program, enabled us to describe more precisely this event responsible for the synthesis of the fusion *PCDH15*-*LINC00844* transcript. Indeed, the 5′ end of the inversion occurred between two highly homologous HERVH elements in inverse orientation within the *PCDH15* intron 13 and the intergenic *BICC1*-*LINC00844* sequence. Unfortunately, none of the reads aligned to the *PCDH15* intron 13 sequence included the 3′ end junction. On the other hand, this genomic sequencing approach allowed a better characterization of the large maternally inherited deletion of E18–26, which could be defined as the c.2092-2569_3502-14202delinsCAC variation. Sanger sequencing of the PCR fragments encompassing these two genomic abnormalities confirmed these results (Figures 4A,B).

In order to fully characterize the large inversion, a long-range PCR with primers expected to amplify the *BICC1*/*PCDH15* junction was then carried out; the resulting 11 kb amplified product was sequenced with the MinIon system and the HERVH junction sequence was validated with the Sanger method (Figure 4C). Due to high homologies between the two HERVH sequences implicated in the inversion junctions, genomic locations of divergent nucleotides (Figure 4D) were used in accordance with the Human Genome Variation Society (HGVS) 3′ rule to describe this SV as the hg38:chr10:g.[(54170766_54170947)_(54171162_54171211)del;(5 4171162_54171211)_(58862555_58862736)inv;(58862291_588623 40)_(58862555_58862736)dup] variant.

## Discussion

Identifying the causal gene responsible for Usher syndrome in a patient will help to provide an appropriate genetic counseling for the family. Also, while cochlear implants improve the hearing function of USH1 patients, there is no treatment for vision loss. In this context, development of different therapeutic strategies including gene therapy is essential. A key element is the identification of the causal gene for each patient, which implies development of new molecular approaches for unresolved cases as reported in this study.

We describe here the first large paracentric inversion implicated in Usher syndrome. This work was initiated by RT-PCR analyses and the identification of the fusion transcripts *PCDH15*-*LINC00844* and *BICC1*-*PCDH15.* Afterward, we took advantage of long-read sequencing technology and were able to decipher the complexity of the inversion, demonstrating the implication of HERVH internal regions and the presence of a segmental duplication associated with a deletion. Importantly, this analysis revealed that neither *BICC1* (MIM#601331; implicated in autosomal dominant renal dysplasia) nor *LINC00844* (ENSG00000237949, Entrez: 100507008, a RNA gene recently described as a regulator of prostate cancer cell and invasion [Lingadahalli et al., 2018]) were disrupted by the inversion.

Even if balanced SVs, as large inversions, are undoubtedly rare events in Mendelian diseases, they are still underestimated as they often remain unexplored in diagnostic laboratories. Indeed, the short-read technology does not handle homologous interspersed repeat elements like HERV (around 3% of the human genome and high homology between copies) which are considered as ideal substrates for non-allelic homologous recombination. Recently, several studies have highlighted the advantages of long-read sequencing, including SV characterization in medical genetics (Mantere et al., 2019, for a review), and our results further emphasize this fact. With a high error rate and a still high sequencing cost, the long-read technology cannot be used routinely in a clinical context, but it definitely offers an interesting approach for unresolved patient cases.

In conclusion, this work describing the first ever large inversion in chromosome 10 leading to Usher syndrome extends the mutational spectrum of the *PCDH15* gene and emphasizes the importance of considering the identification of balanced SVs for exhaustive molecular diagnostics.

## Data Availability Statement

The datasets presented in this study can be found in online repositories. The names of the repository/repositories and accession number(s) can be found below: https://figshare.com/, https://doi.org/10.6084/m9.figshare.12137313.v1.

## Ethics Statement

The studies involving human participants were reviewed and approved by the Montpellier University Hospital (CHU Montpellier, Montpellier, France) as part of a molecular diagnostic activity and were performed in accordance with the French law on bioethics: “loi de bioéthique”, revised 7 July 2011, number 2011–814. The authorization number given by the Agence Régionale de la Santé (ARS) is LR/2018-N°3005-RT 34-16-16. The patients/participants provided their written informed consent to participate in this study. Written informed consent was obtained from the individual(s) for the publication of any potentially identifiable images or data included in this article.

## Author Contributions

CV, A-FR, JP, and FP contributed to the conception and the design of the study. CV, VF, FD, AL, GG-G performed the experiments. CV, JP, FD, AL, DB, and GG-G analyzed and interpreted the data. CB and IM provided patients’ samples and clinical data. CV, CB, and IM wrote the first draft of the manuscript. A-FR, JP, FP, and MK revised the manuscrpit. All authors contributed to the article and approved the submitted version.

## Conflict of Interest

The authors declare that the research was conducted in the absence of any commercial or financial relationships that could be construed as a potential conflict of interest.

## Footnotes

1 http://LOVD.nl/PCDH15

2 https://www.biap.org/

3 http://varnomen.hgvs.org/

4 https://databases.lovd.nl/shared/

5 http://gnomad.broadinstitute.org

6 http://browser.1000genomes.org

7 http://exac.brodinstitute.org
