# Supplementary material for: A 4.6 Mb Inversion Leading to PCDH15-LINC00844 and BICC1-PCDH15 Fusion Transcripts as a New Pathogenic Mechanism Implicated in Usher Syndrome Type 1
Source: Front Genet. 2020 Jul 2;11:623. doi: 10.3389/fgene.2020.00623 (PMC7343966; doi:10.3389/fgene.2020.00623)
Supplement: Supplementary file 1 [file Data_Sheet_1.PDF]

Supplementary Tables

Table S1. S1 to S34 *PCDH15* MLPA probes

Probe number	SALSA MLPA probe	Exon targeted
S1	08746-L113227	1
S2	08747-L11148	1
S3	08748-L08788	2
S4	08749-L08789	2
S5	08750-L08790	3
S6	08751-L08791	4
S7	08752-L11967	5
S8	08753-L08793	6
S9	08754-L08794	7
S10	08755-L08795	8
S11	08756-L08796	9
S12	08757-L08797	10
S13	08758-L08798	11
S14	08759-L11326	12
S15	08760-L11318	13
S16	08761-L08801	14
S17	08762-L08802	15
S18	10733-L11966	16
S19	08764-L11316	17
S20	08765-L08805	18
S21	08766-L08806	19
S22	08766-L11764	20
S23	08768-L11325	21
S24	08769-L11317	22
S25	08786-L08810	23
S26	08787-L11328	24
S27	08787-L11328	25
S28	11542-L12284	26
S29	08790-L11322	27
S30	08791-L08815	28
S31	08792-L08816	29
S32	08793-L08817	30
S33	08794-L08818	32
S34	08795-L08819	33

Table S2. Sequences of primers for RT-PCR reactions

Amplicon	PCR1 primers (5'-3')	PCR2 primers (5'-3')	Comments
**F1**<br>Exons<br>(2-10)	ggacatgtttagcttcaggg<br>tggtgaaagtctctgtttactgg	acatgtttagcttcagggatc<br>ggaaaaatcgtgggtaatcc	c.55T>G in the paternal inherited allele
**F2**<br>Exons<br>(9-19)	tgaaccccattattgttacgc<br>tgttgaattggtgaacacagg	aatccaagccattgatcagg<br>caatgtccaaaaccttgatgg	At least one primer in the maternal inherited deletion
**F3**<br>Exons<br>(17-22)	tagggaaagcactggacagg<br>cacacgacttgcaggtaatcc	gtggaagaagaagccaatgc<br>gtgtacccttgactgcatcc	
**F4**<br>Exons<br>(20-27)	ttgcactacatccatttacagg<br>tccactacaaatccttcttttcc	gcatttccagaccaagaagc<br>tctcaatgtatactttagctgtattgc	
**F5**<br>Exons<br>(24-CD3)	agtgaacttgccaccaaagg<br>cgattaattgataacaatgtg	ttcgagtccaagctgattcc<br>ggcttcaccgctgtattgtcag	
